# Pre-operative Percutaneous Nephrolithotripsy Characterisation of Kidney Stones with Second-Generation Dual-Source Dual-Energy Computed Tomography

**DOI:** 10.21315/mjms2020.27.5.5

**Published:** 2020-10-27

**Authors:** DK Mella Mohd Ali, Mohd Hafizi Mahmud, Noor Shafini Mohamad

**Affiliations:** 1 Centre of Medical Imaging, Faculty of Health Sciences, Universiti Teknologi MARA (UiTM) Selangor Branch, Puncak Alam Campus, Selangor, Malaysia; 2 Radiology Department, Hospital Tengku Ampuan Afzan Pahang, Pahang, Malaysia

**Keywords:** dual-source computed tomography, chemical composition, kidney stone, percutaneous nephrolithotripsy

## Abstract

**Background:**

The current clinical practice to manage kidney stone requires knowledge of the stone composition. However, it is often difficult to determine the actual stone composition before a stone is operatively removed from the patient. Dual-energy computed tomography (DECT) can predict urinary stone composition, but it is not widely adopted. The purpose of the study was to investigate the use of a second-generation DECT with tin or stannum (Sn) filter for characterising the kidney stones composition.

**Methods:**

Thirty-three kidney stones were scanned ex vivo using a dual-source (DS)-DECT scanner with dual-energy (DE) mode of 80/140 kVp with and without 4 mm Sn filtration. DE ratio was calculated to determine the kidney stones composition (uric acid, calcium oxalate, calcium phosphate and cystine). The median DE ratio of the stones was compared using Wilcoxon signed rank test and the results were further correlated with semi-quantitative Fourier transform infrared (FTIR) spectroscopy analysis using Kendall’s Tau test with *P* < 0.05 deemed to be statistically significant.

**Results:**

Second-generation DS-DECT could significantly discriminate the stones composition with and without Sn filtration (*P* < 0.001). The median DE ratio of uric acid, calcium oxalate and cystine stones were significantly higher with Sn filtration than those without filtration (*P* < 0.05). DECT results revealed significant correlation with FTIR spectroscopy analysis (*r* = 0.716, *P* < 0.001). DECT with Sn filtration showed increased performance (100% sensitivity, 0% specificity) than those without filtration (48.5% sensitivity, 0% specificity) in the detection of the kidney stone subtypes.

**Conclusion:**

In the second-generation DECT with additional Sn filtration, DECT has shown a significant performance in characterising and discriminating the kidney stone composition. This may improve diagnostic and therapy management in kidney stones cases.

## Introduction

The most common organ for formation of stone in the human body is in the kidneys; kidney stone is known to be associated with aging population and dietary habits (1, 2). In Malaysia, sex and age are the determining factors of prevalence of kidney stones: male have higher tendency to form kidney stones than female (57.5% male, 42.5% female) and older people are more prone.

Calcium is found to be the most common component of kidney stone among Malaysians (3, 4), usually in the form of calcium oxalate or calcium phosphate. Other types include struvite stones, uric acid stones and cystine stones.

The recommended imaging modalities for the diagnosis of kidney stone, according to Urological Association of Asia (UAA) and European Guidelines, are plain radiography, ultrasound and non-contrast computed tomography (NCCT) (5, 6). Although NCCT can visualise (7) and predict stone clearance rate (8, 9), NCCT is unable to characterise stone composition (10). Pre-operative determination of kidney stone composition is essential for selecting efficient treatment and preventive management (5, 11).

Kidney stones such as brushite (a unique calcium phosphate stone), calcium oxalate monohydrate and cystine should be treated by pre-operative percutaneous nephrolithotripsy (PCNL) or retrograde intrarenal surgery (RIRS) (12). Dual-energy computed tomography (DECT) is a reliable method to determine kidney stone composition in vivo (13–16) and in vitro (17–20). However, results were inconsistent in terms of reliable differentiation of calcium to other compositions because confidence intervals overlapped, attenuation thresholds to define a calcium stone varied, and scanner settings and methods for chemical analysis differed markedly.

The recent advancement of CT in the management of stone disease has reduced the variability of the results, thus decreasing long term complications and rate of recurrences (11, 21).

Additional filtration has the advantage of shaping the energy spectra between high and lower tube, improving the material identification (7). The addition of Sn filter at the higher tube (140 kVp) may not be essential for the simple demarcation between uric acid and non-uric acid stones, nevertheless the added Sn filter plays a significant role to detect complicated stones such as calcium oxalate and calcium phosphate (22). Therefore, this study investigates the kidney stone characterisation with second-generation DECT in a phantom model. Findings were compared to semi-quantitative Fourier transform infrared (FTIR) spectroscopy analysis, a reference standard for chemical kidney stone analysis.

## Methods

### Stone Sample Collection and Phantom Model

All renal stones were not linked to the patients; hence the requirement for consent was waived. Thirty-three renal stones were collected and analysed with second-generation DS-DECT and semi-quantitative FTIR spectroscopy analysis.

The stones were taken from the surgical procedure, PCNL by a urologist. The stones were placed at room temperature, each in a separate container filled with water gel to prevent the stones from drying. The stones collected were required to comprise 85% or more component of interest. The size of the stones were 4 mm or larger in diameter. This will ensure that the stones consist of one chemical composition and stone size bigger than 4 mm to obtain accurate attenuation values.

**Figure 1:**
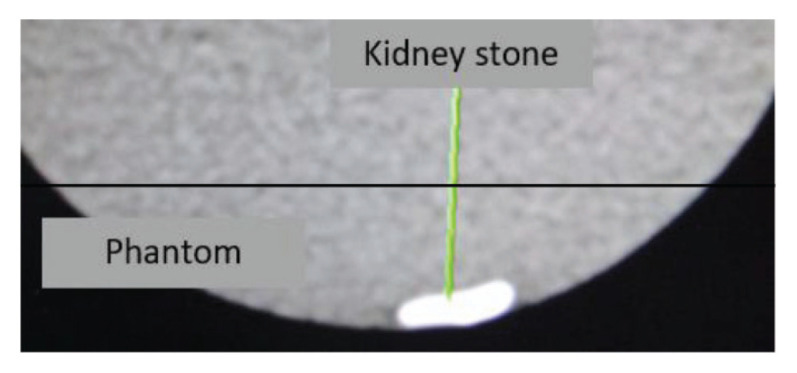
CT image of a kidney stone embedded in a jelly phantom measuring 6 mm largest diameter was derived by the software

The stones were embedded in a jelly phantom tank with a volume of 184 mm × 144 mm × 368 mm (Figure 1). The phantom was made of water, animal protein and iodine. A total of 21.4 g of jelly was mixed with 3 L of water equivalent to the attenuation of water (10 HU–20 HU at 140 kVp), and the stones were subsequently scanned using DECT.

### Dual-Source DECT and Acquisition Protocol

Dual-source (DS) system, 256-slice DS-DECT (Somatom Definition Flash, Siemens Medical Solutions, Forchheim, Germany) equipped with two detector arrays with different sizes and two X-ray tubes orthogonal to each other within the gantry was used for the analysis.

The scanner was operated at 80 kVp and 140 kVp simultaneously with a scan field of view of 50 cm in tube A and a scan field of view of 33 cm in tube B. Scans were performed using DE kidney stone protocol with and without 4 mm Sn filter based on the manufacturer settings (128 mm × 0.6 mm collimation, 0.5 gantry rotation time and 0.7 pitch with spiral mode) (Table 1). The automatic exposure control (AEC) modulation technique and attenuation-based tube current modulation (CareDose4D, Siemens Healthcare) were switched on to suit with the clinical routine.

### Stone Material Characterisation

**Figure 2:**
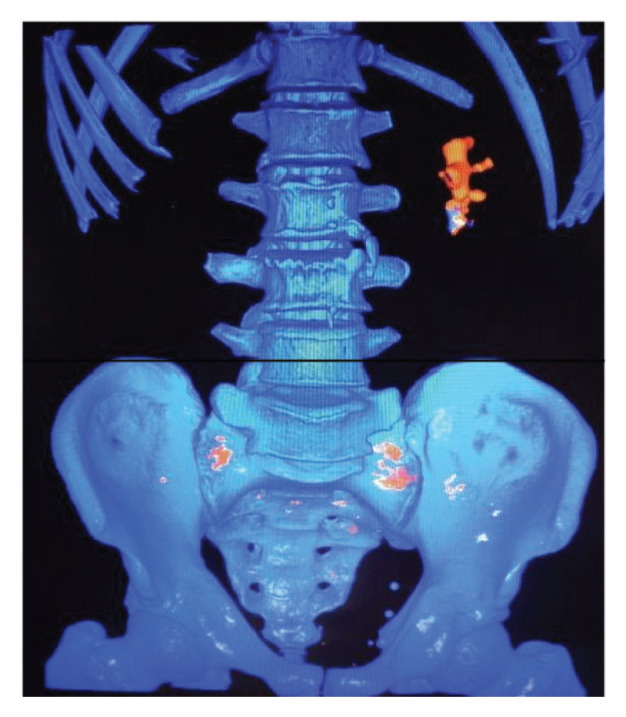
An example of colour-coded image produced by Renal Stone Kidney Analysis software (SyngoVia VA20B, Siemens Healthcare); uric acid stone (red) and non-uric acid stone (blue) in vivo

Image series for 80 kVp and 140 kVp were reconstructed at a slice thickness of 0.75 mm with 0.04 increments. Both image series were acquired in the axial, coronal and sagittal planes using a standard soft tissue kernel (Q30f) and dedicated Renal Stone Analysis software (SyngoVia VA20B, Siemens Healthcare) (Figure 2). Measurements of DE ratio and average HU for each stone were recorded by manually placing a circle using a circle tool at region of interest (ROI). The circle was drawn smaller than 50% of each stone diameter to avoid the partial volume artefact along the periphery. The minimum HU unit in this setting was 200.

The attenuation profile of the stone was presented as plot on a graph representing low and high peak kilovoltage as *x* and *y* axes, respectively. The DE ratio was obtained by dividing the attenuation value of the stones at 80 kVp by its attenuation value at 140 kVp, whilst the DE number was obtained by subtracting the low energy CT from high energy CT. The software displayed the stones in red and blue colours according to colour map (red for uric acid stone, blue for non-uric acid stone) (Figure 2).

### Chemical Analysis of Stone Composition

The stones were washed with distilled water to remove loose debris such as blood and mucous and air-dried (20 °C–22 °C) for 24 h. A sample of stone was mixed with 100 mg potassium bromide and pulverised by mortar and pestle to develop a homogenous fine powder.

Then, a small amount of fine powder of the renal material and potassium bromide were blended under pressure using a specially evacuated dye to create a pallet. The pallet was placed on a sample holder for FTIR spectroscopy analysis. The spectrum of the sample was recorded in the spectrum FTIR spectroscopy analysis device (PerkinElmer). The results were compared with that of DS-DECT.

### Statistical Analysis

The median and interquartile range (IQR) were calculated for the measured DE ratio. Wilcoxon signed rank test was performed to evaluate DE ratio of stone compositions between scanning protocols without and with filter. The DS-DECT results were further correlated with FTIR spectroscopy analysis using Kendall’s Tau analysis. The statistical analysis was performed using SPSS (PASW version 26.0) with *P* < 0.05 deemed significant.

## Results

All the stones with diameter range of 4 mm–10 mm (*n* = 33) were scanned using DS-DECT with and without 4 mm Sn filter. There were 10 uric acid stones and 23 non-uric acid stones. Of the non-uric acid stones, 16 calcium oxalate, 3 calcium phosphate and 4 cystine were identified with and without Sn filter, and CTDI_vol_ were 1.00 mGy and 1.84 mGy, respectively.

**Figure 3:**
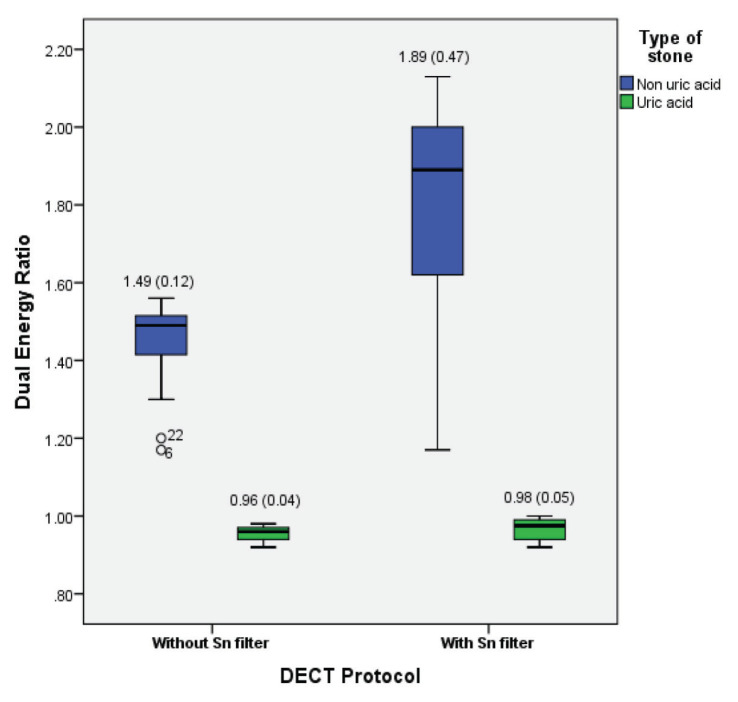
Box whisker plot with first and third quartiles (boxes) of median DE ratio in uric acid and non-uric acid stones. Each of the DE ratio were plotted against DECT protocol (without and with Sn filter)

Box and whisker plots show that both protocols were able to separate all uric acid and non-uric acid stones, with Sn filter protocol showing greater median and absolute value range (Figure 3). Higher DE ratio for non-uric acid and uric acid stones were observed in DECT with Sn filter protocol (median 1.89 [IQR 0.47] and median 0.98 [IQR 0.05], respectively) as compared to DE ratio of those in DECT without Sn filter (median 1.49 [IQR 0.12] and median 0.96 [IQR 0.04], respectively). The median DE ratio for non-uric acid containing stones was substantially higher with Sn filter as compared to without Sn filter.

**Figure 4:**
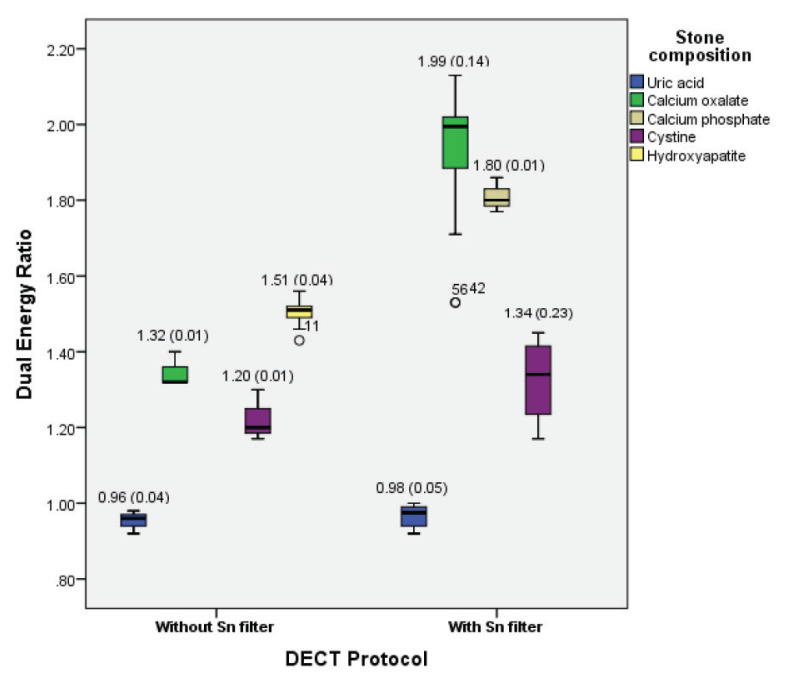
Median DE ratio of uric acid and various compositions of non-uric acid stones versus DECT protocol (without and with Sn filter)

DE ratio for uric acid, calcium oxalate and cystine were significantly higher with Sn filtration protocol as compared to without Sn filtration protocol (*P* < 0.05) (Table 2). Among those compositions, calcium oxalate showed the substantial DE ratio variance of 33.7%. Sufficient separation was observed in both without and with additional Sn filtration for non-uric acid stones, with additional Sn filtration shows larger separation (Figure 4). The results showed significant overlap in the DE ratio between calcium oxalate/calcium phosphate and hydroxyapatite.

Kendall’s Tau correlation shows significant correlation between DECT and FTIR spectroscopy analysis (*r* = 0.716, *P* < 0.001). All stones characterised as uric acid by FTIR spectroscopy analysis were correctly identified as uric acid stones by DS-DECT, with and without Sn filter (*n* = 10). None of uric acid and non-uric acid stones were misclassified by DS-DECT with Sn filter.

However, 13 calcium oxalate stones, three calcium phosphate and one cystine were misclassified as hydroxyapatite by DS-DECT without Sn filter. All 10 uric acid stones and 3 out of 4 cystine stones were visible at DECT without filtration (< 1.5 DE ratio).

DS-DECT with Sn filtration could identify all 33 stones correctly with 100% sensitivity and 0% specificity relative to that of FTIR spectroscopy analysis. Whilst DS-DECT without Sn filtration recognised 17 out of 33 stones and showed 48.5% sensitivity and 0% specificity. Table 3 provides the detailed overview of the results obtained by DECT and FTIR spectroscopy analysis.

## Discussion

This study was conducted to evaluate the ability of the second-generation DS-DECT with 4 mm Sn filter to precisely represent the composition of uric acid stones. It was found that there was an improvement in spectral separation as a result of increased DE ratio using Sn filtration. With high spectral separation, the uric acid stones markedly differed from non-uric acid ones in the two protocols. The data from the study is consistent with the results in the literature (7) which suggest that the use of Sn filter in DECT could significantly increase differentiation between uric and non-uric acid stones.

Moreover, the present study shows calcium-based stones (calcium oxalate and calcium phosphate) as significantly depicted composition when using Sn filtration protocol which have not been addressed in the previous literatures. DECT with Sn filtration has a significant role for discrimination of non-uric acid stone compositions particularly with small effective atomic number (Z*_eff_)* variation such as calcium-based stones.

Calcium stones (72.6%) are the most common type of stone in Malaysian East Coast region, followed by uric acid (16.4%) and unidentified stones (10.9%) (4). The trend is similar with other Asian countries and higher compared to other parts of the world (5).

Knowledge regarding stone composition is important and has high clinical relevance for decision of treatment strategies to optimise management of patient with kidney stone (13, 18, 21, 23). Calcium-based stones often require PCNL and are less likely to be successful with shock wave lithotripsy (SWL) (5, 24). Clinical management of the uric acid stone is commonly based on the chemolysis to alkalise the urine and reduce the crystallisation phase (21). However, if the chemolysis failed, the SWL is amenable.

In Malaysia, the clinical management of kidney stones follows UAA guidelines (5). However, to our knowledge, the composition of the kidney stones is not identified prior to operative procedure; only selective type of patients are arranged for stone composition analysis post-operatively. This is usually reserved for pediatric patients.

For the first generation DECT, 64-detector row CT (Somatom Definition; Siemens Medical Solutions, Forchheim, Germany) without an additional filtration, the kidney stones were characterised using Matlab (version 7.4, and Image Processing Toolbox, version 5.4; MathWorks, Natick, Mass) for identifying cystine, struvite and brushite, calcium oxalate and uric acid (25).

The current study utilised second-generation 256-slice DS-DECT (Somatom Definition Flash, Siemens Medical Solutions, Forchheim, Germany) with added filtration to characterise uric and non-uric acid stones. The non-uric acid stones were further divided into different compositions: calcium oxalate, calcium phosphate and cystine. Renal stone analysis displayed the image of the stone with two colours; red is uric acid stone and blue is non-uric stone (Figure 2).

The added Sn filter is important for the non-uric acid stone subtypes due to a slight overlap between the calcium oxalate, calcium phosphate and hydroxyapatite. Presence of added filtration in the DS DE increases the spectral separation between the stones like in uric and non-uric stones (19). Without Sn filtration, the calcium oxalate and calcium phosphate could not be differentiated clearly from hydroxyapatite. These data suggest the photon energy threshold for 80/140 kVp protocol has caused linear attenuation coefficients to be overlapped by less than 1 standard deviation. This is important not only because of low threshold level, but also because of higher concentration of hydroxyapatite, potentially no less than 808.5 mg/cm^3^ (26).

The additional Sn filtration has a significant role to characterise calcium-based stones as both calcium oxalate and calcium phosphate were able to be identified using this protocol but not in those without filtration as shown in Table 3. Of total 16 calcium oxalate stones, three were detected accurately by both DECT with and without filtration at DE ratio of 1.53 versus 1.32 (*n* = 2) and 1.71 versus 1.40 (*n* = 1), respectively. DECT without filtration fails to detect calcium phosphate (*n* = 3) with DE ratio of 1.43, 1.46 and 1.48. As indicated with a low sensitivity of 48.5%, DECT without filtration would not be able to identify kidney stones accurately.

Nevertheless, the DECT with filtration is possible to circumscribe DE ratio for calcium oxalate from 1.53 to 2.13 and calcium phosphate from 1.80 to 1.86 under in vitro conditions at 80/Sn140 kVp. These increased ratios allow for the detection of 85% pure components stone. Detection of > 85% of calcium components influences attenuation significantly and it is, therefore, amenable to PCNL.

The distribution of brushite and struvite stones is not equal when only 2% of pure struvite stone could be found clinically. On an improved sensitivity of 100%, this would increase the likelihood of DECT with filtration for detecting the presence of kidney stones subtypes accurately. Although urologists are supposed to managed the kidney stone disease by following the UAA guidelines, several issues existed, for example, asymptomatic patient with kidney stone was seldom advised on stone recurrence prevention (27).

For all the four types of kidney stones, the result shows that median DE ratio with Sn filtration was higher compared to DECT without Sn filtration. Sn filter was applied on high tube voltage (Sn140 kV). Sn filter tends to attenuate lower X-ray energies from the high tube voltage and harden the spectrum. Therefore, its usage has improved spectral differentiation between the stones (16, 28). It is known that both with and without filter can separate the urid acid from non-uric acid stones (29). The urid acid stones showed hypodense attenuation between 300 HU and 600 HU at both tube voltages (80/Sn140 kVp) (30), followed by cystine (450 HU–1500 HU), calcium phosphate (920 HU–1800 HU) and hyperdense attenuation calcium oxalate (300 HU–2500 HU). For future work, a micro-CT approach could be used to further visuali*s*e the homogeneous and heterogeneous mixtures of each of the stones at a higher resolution.

The radiation dose of DECT with filtration is lower compared to without filtration (1.00 mGy versus 1.84 mGy). Comparing the second-generation DECT with first generation, the radiation dose is much lower up to 94.4%, particularly for phantom study (30) and up to 40% decrease in radiation dose in DECT compared to single-energy CT for patients (31). Although, the current study was carried out on a phantom using second-generation DECT, the protocol applied was a routine to mimic a clinically relevant situation.

The current study has some limitations that have to be considered. Firstly, only a few stones were found to be in the mixed compositions. The mixed stone is composed of different substances and more common compared to pure stone. In a study, the composition of stone at the central nucleus may be different from the composition at the outer layers due to many factors that cause stone growth (32). Secondly, the 33 stones are not equally distributed in size; and the measurements represent a small sample size. A larger amount of stones could provide more precise values.

## Conclusion

In conclusion, on the basis of the results of this ex-vivo study, second-generation DECT is a useful technique to characterise the compositions of uric acid and non-uric acid stones. DECT with Sn filtration shows higher DE ratio than DECT without Sn filtration, which significance for discrimination of non-uric acid stones compositions particularly with small Z*_eff_* variation. Second-generation DECT with Sn filtration is a potential non-destructive tool in pre-operative analysis for characterisation of renal stone compositions, conforming to the post-operative FTIR spectroscopy analysis for predicting the choice of treatment, thus improving patient management in kidney stone cases.

## Figures and Tables

**Table 1:** DS-DECT scanning parameters

Scan parameter	Without Sn filter	With Sn filter
Tube potential (kVp)	80 and Sn140	80 and 140
Effective mAs	14/264	12/192
Collimation (mm)	128 × 0.6	128 × 0.6
Rotation time (s)	0.5	0.5
Pitch	0.7	0.7
Automatic exposure control	Yes	Yes
CAREDOSE	Yes	Yes
Volume CT dose index (mGy)	1.84	1.00

**Table 2:** Median and interquartile range (IQR) of DE ratio for the uric acid and three non-uric acid stone subtypes

Types of stones	*n*	Without Sn filter	With Sn filter	DE ratio difference (%)	*P*-value
Uric acid	10	0.96 (0.04)	0.98 (0.05)	2.0	0.04^*^
Calcium oxalate	16	1.32 (0.01)	1.99 (0.14)	33.7	< 0.001^*^
Calcium phosphate	3	1.46 (0.05)	1.80 (0.09)	19.0	0.10
Cystine	4	1.20 (0.01)	1.34 (0.23)	10.4	0.02^*^

Note:

The asterisk^*^ indicates significant result (< 0.05)

**Table 3:** Type of renal stones predicted with DECT and FTIR spectroscopy analysis

Stone	CT predicted stone with filter	CT predicted stone without filter	FTIR spectroscopy analysis result
1	CaO	HA	CaO
2	CaO	HA	CaO
3	CaO	HA	CaO
4	CaO	HA	CaO
5	CaO	HA	CaO
6	CYS	CYS	CYS
7	CaO	HA	CaO
8	CYS	CYS	CYS
9	CaO	CaO	CaO
10	CaP	HA	CaP
11	CaP	HA	CaP
12	CaP	HA	CaP
13	CaO	HA	CaO
14	CaO	HA	CaO
15	CaO	HA	CaO
16	UA	UA	UA
17	UA	UA	UA
18	UA	UA	UA
19	UA	UA	UA
20	UA	UA	UA
21	UA	UA	UA
22	CYS	CYS	CYS
23	CaO	CaO	CaO
24	UA	UA	UA
25	UA	UA	UA
26	UA	UA	UA
27	UA	UA	UA
28	CYS	HA	CYS
29	CaO	CaO	CaO
30	CaO	HA	CaO
31	CaO	HA	CaO
32	CaO	HA	CaO
33	CaO	HA	CaO

Notes: UA = uric acid; CaO = calcium oxalate; CaP = calcium phosphate; CYS = cystine; HA = hydroxyapatite
